# Health outcomes, education, healthcare delivery and quality – 3042. Quality of life in children with atopic dermatitis and its relationship with disease severity

**DOI:** 10.1186/1939-4551-6-S1-P215

**Published:** 2013-04-23

**Authors:** Vivek Rungta

**Affiliations:** 1 Dermatology, Gauhati Medical College and Hospital, Guwahati, India

## Background

Atopic Dermatitis (AD) is a chronic relapsing skin disease occurs due to exogenous and endogenous cause.

It has a tremendous psychological impact on individual’s life. It can interfere with the mental stability, social and emotional adjustment and Quality of Life (QOL)

This study aims to document the Quality of Life in children suffering from Atopic Dermatitis and its relationship with disease severity.

## Methods

A total of thirty children aged 5-16 years suffering from AD (diagnosed by Hanifin and Rajka criteria) and thirty age, sex matched control group were included in the study.

The Children’s Dermatology Life Quality Index (CDLQI) questionnaire was used to quantify the impact of Atopic Dermatitis on Quality of Life.

Eczema severity was assessed by using six area six sign Atopic Dermatitis Severity Score Index.

Statistical analysis was done by Spearman correlation coefficient by using Instat3 software to compare CDLQI with control and disease severity.

## Results

Quality of Life is hampered more in Atopic children than control.

Also it has been found thatQ_1_, Q_2_, Q_5_, Q_6_, Q_9_, Q_10_were elevated; which imply children’s symptoms, feelings, leisure, sleep and treatment were affected by the impact of the disease.

Children’s Quality of Life has shown a significant positive correlation with disease severity (r=0.8526, p<0.0001)

## Conclusions

CDLQI can be used as an extra measure of disease assessment in the management of Atopic Dermatitis. Though it is not a life threatening disease, its impact on Quality of Life should not be overlooked while evaluating this disease.

